# Supplementary material for: Exploring the relationship between regional tourism development and land use efficiency: A case study of Guangxi Zhuang Autonomous Region, China
Source: PLoS One. 2024 Jan 25;19(1):e0297196. doi: 10.1371/journal.pone.0297196 (PMC10810482; doi:10.1371/journal.pone.0297196)
Supplement: Attachment — Submitted filename: Respond to Reviewers.docx [file pone.0297196.s003.docx]

Response to Reviewer Comments

First of all, thank you for your valuable comments on this manuscript. We have carefully revised it based on your comments. We have revised them one by one based on your comments and marked them with the "**Track Change**s" function in Microsoft Word. Because different people may see different line numbers when using the word format version under "Track Changes" mode. **the line number in the response letter refers to the line number in PDF format version.** In the following reply report, point-by-point answers to the comments are listed. We hope this revised manuscript has addressed your concerns, and look forward to hearing from you.

**Point 1:** Please ensure that your manuscript meets PLOS ONE's style requirements, including those for file naming. The PLOS ONE style templates can be found at https://journals.plos.org/plosone/s/file?id=wjVg/PLOSOne_formatting_sample_main_body.pdf and

https://journals.plos.org/plosone/s/file?id=ba62/PLOSOne_formatting_sample_title_authors_affiliations.pdf

**Response 1:** **Thank you for your reminder. according to the style requirements of PLOS ONE, I have revised the manuscript.**

**Point 2:** We note that the grant information you provided in the ‘Funding Information’ and ‘Financial Disclosure’ sections do not match.

When you resubmit, please ensure that you provide the correct grant numbers for the awards you received for your study in the ‘Funding Information’ section.

**Response 2: Thank you for your reminder. I have verified that there was an error in the 'Financial Disclosure' section and that 'Funding Information' is correct. This article was financially supported by’** **the National Office for Philosophy and Social Sciences: Research on Tourism Cultural Space Production and Social Governance Innovation in southwest minority traditional villages (20BJY210)’.**

**Point 3:** We note that you have stated that you will provide repository information for your data at acceptance. Should your manuscript be accepted for publication, we will hold it until you provide the relevant accession numbers or DOIs necessary to access your data. If you wish to make changes to your Data Availability statement, please describe these changes in your cover letter and we will update your Data Availability statement to reflect the information you provide.

**Response 3: Thank you for your reminder.** **Yes, I will provide repository information for my data.** **All data files are available from the Figshare database (doi: https://doi.org/10.6084/m9.figshare.24126672)**

**Point 4:** We note that [Figures 1 and 4] in your submission contain [map/satellite] images which may be copyrighted. All PLOS content is published under the Creative Commons Attribution License (CC BY 4.0), which means that the manuscript, images, and Supporting Information files will be freely available online, and any third party is permitted to access, download, copy, distribute, and use these materials in any way, even commercially, with proper attribution. For these reasons, we cannot publish previously copyrighted maps or satellite images created using proprietary data, such as Google software (Google Maps, Street View, and Earth). For more information, see our copyright guidelines: http://journals.plos.org/plosone/s/licenses-and-copyright.

**Response 4: Thank you for your reminder.** **I have provided the source for the images in Fig 1 and Fig 4. Which is “Note: Based on the standard map production of the National Natural Resources Department's Standard Map Service website (GS(2019)1822), the base map boundaries have not been modified”.** **（http://bzdt.ch.mnr.gov.cn/download.html?searchText=GS(2019)1822）**

**Point 5:** Please review your reference list to ensure that it is complete and correct. If you have cited papers that have been retracted, please include the rationale for doing so in the manuscript text, or remove these references and replace them with relevant current references. Any changes to the reference list should be mentioned in the rebuttal letter that accompanies your revised manuscript. If you need to cite a retracted article, indicate the article’s retracted status in the References list and also include a citation and full reference for the retraction notice.

**Response 5: Thank you for your reminder. I have verified that all references in this article are complete and correct and have not been retracted**

**Point 6:** **Revision comments in the manuscript**

**a:”** Its importance to land use.” Is this an incomplete sentence?

**Response 6(a): It has been revised. The correct sentence is “This also highlights the importance of land use.”**

**b:”** Data Development Envelopment Analysis.**”** Is this the correct term? Data Development or Data Envelopment Analysis?

**Response 6(b): It has been revised. The correct word is** **“Data Envelopment Analysis”.**
