# Supplementary material for: Exploring the relationship between regional tourism development and land use efficiency: A case study of Guangxi Zhuang Autonomous Region, China
Source: PLoS One. 2024 Jan 25;19(1):e0297196. doi: 10.1371/journal.pone.0297196 (PMC10810482; doi:10.1371/journal.pone.0297196)
Supplement: Attachment — Submitted filename: Respond to Reviewers2.docx [file pone.0297196.s004.docx]

Response to Reviewer#3 Comments

First of all, thank you for your valuable comments on this manuscript. We have carefully revised it based on your comments. We have revised them one by one based on your comments and marked them with the "**Track Change**s" function in Microsoft Word. Because different people may see different line numbers when using the word format version under "Track Changes" mode. **the line number in the response letter refers to the line number in PDF format version.** In the following reply report, point-by-point answers to the comments are listed. We hope this revised manuscript has addressed your concerns, and look forward to hearing from you.

**Point 1:** 1. Abstract: This section is written descriptively. It is suggested to include the study findings in a quantitative way.

**Response 1: Thank you for your reminder.** **I made adjustments to the abstract, and described the conclusions in a quantitative manner.**

**“There is a reciprocal causation relationship between the Regional tourism development level and land use efficiency in Guangxi, with significant levels of 0.005 and 0.034 respectively, indicating high credibility. This indicates that there is a mutual promotion and interaction between the two, which rely on and drive each other, promoting the joint sustainability of tourism development and land use efficiency”**

**“The tourism development level is greatly influenced by itself, with impact values all above 0.99. At the same time, land use also has a significant self-impact, with impact values all above 0.87.”**

**Delete”** **mutually promote and interact. The two push and rely on each other to cement their relationship. “ , ”Tourism development level and land use efficiency are greatly influenced by themselves”**

**Point 2:** Introduction: (a). This section was written too short and is missing important aspects. (b). The novelty of the study is not properly outlined. (c). Organization of the study is also missing at the end of this section.

**Response 2: Thank you for your reminder.** **The introduction section has been added with the following paragraphs**

**“Tourism development and construction require a large amount of land for building tourist attractions, hotels, and other related facilities. If the land use efficiency in the research area for tourism development is not high, it is very likely to damage the land quality and other useful values of the land. Therefore, studying the relationship between the tourism development level and land use efficiency is of great value.”**

**“According to the national policy of 'Three Zones and Three Lines', Guangxi has formulated its own 'Three Zones and Three Lines' policy, which aims to improve the national land spatial planning, enhance the quality and value of land utilization, and ensure the reasonable development of cultural and tourism industries.”**

**“Government policies will continuously address various land use issues that arise in the development of tourism.”**

**“Limited land resources have become an important element in the development of tourism.”**

**“As an important component of tourism resources and a carrier for tourism development, land resources cannot be ignored. Therefore, in the study of the entire tourism development process in Guangxi, our research team further investigated the utilization of tourism land resources.”**

**Delete”** **Limited land resources have always been an essential element in the competition for use in ancient and modern times and are now a necessary part of tourism development.”**

**Point 3:** Literature Review: This section should be divided into two parts (a). The first section must be based on the theoretical foundation followed by the empirical review directly or indirectly related to this study (b). Include up-to-date literature as most of the studies reviewed in this section are old. (c) Furthermore, it is recommended to find out the research gap that this study fills at the end of this section. Please consult papers https://doi.org/10.3390/en14133923; https://doi.org/10.1007/s10668-020-01023-2; https://doi.org/10.1007/s10668-022-02194-w for improving and develop this section.

**Response 3: Thank you for your reminder.** **The literature review section has been added with the following paragraphs, and references have been made to the literature you recommended.**

**“Tourism, as a typical form of land use activity, is one of the industries that rely on land resources. Land resources are the basic conditions and important resource support for the development of tourism.”**

**“Dilawar Khan used SFA to study the relationship between energy efficiency and ecological footprint and believed that efficient utilization of energy resources, as well as investment in agriculture, are necessary for a sustainable environment.”**

**“Meanwhile, Professor Dilawar Khan also studied the impact of technological innovation on energy efficiency”**

**“Zainab Bibi studied the technical and environmental efficiency of the agriculture sector in South Asia and suggested that the agriculture sector should prioritize collaboration in research and development.”**

**Delete”** **The high demand for land in early growth, the expansion of urban areas in pursuit of the speed of development, and the relatively high level of waste associated with the increased consumption of land by urban development have once again led to a reflection by scholars and politicians.”**

**Delete “Meng Tao used the SFA method to study the regional differences in China's tourism industry. The study shows that the regional differences are significant, but the difference value is shrinking.”**

**Delete “The urban land use efficiency was calculated by the SFA method. Bao Xinzhong believes that the problem of urbanization lies in the contradiction between man and land. The solution to this problem is also the need to realize the sustainable development of cities and [ ] the need to recognize tourism development.”**

**Point 4:** Methodology: (a). It is recommended to discuss the significance of each variable included in this study/model (c). Furthermore, the econometric model that examines the impact of independent variables on the dependent variable should be justified (d). It is suggested to discuss how to address different econometric issues in the model.

**Response 4: Thank you for your reminder.** **The meaning of omitted variables has been added, and other econometric issues have been discussed.**

**“W_i_, X_i_ respectively represent the calculation weights of indicators and the level of tourism development in year I”**

**“The PVAR model can analyze the interaction between endogenous variables in panel data, which not only effectively solves the problem of individual heterogeneity, but also fully considers the individual and time effects. This model can solve the econometric problems involved in different fields. Therefore”**

**For this model, variable stationarity has been tested using two methods, Granger causality and stationarity test, in sections 5.2 and 5.1**

**Point 5:** Data analysis and research results: (a). It is recommended to name this section “Results and Discussion” (b).It is recommended to correlate your study with previous studies conducted.

**Response 5: Thank you for your reminder.** **I have changed this section to 'Results and Discussion'.** **and the following content has been added.**

**”** **In previous studies, scholars have found that the grasslands and unused lands in Guangxi have gradually decreased, and there has been a conversion between different types of land use, which has led to a decline in land quality”**

**“We should promote the high-quality development of tourism and the sustainable use of land in Guangxi.”**

**“This has also become the current research and development direction for scholars and tourism companies.”**

**Point 6:** It is recommended to include “Future Research Direction” at the end of the manuscript.

**Response 6: Thank you for your reminder.** **I have added future research directions.”** **This paper explores the correlation and degree of interaction between tourism development level and land use efficiency through research. In future studies, the focus will be on the land planning and utilization of rural tourism, optimizing the rural industrial structure, and researching the demand for tourism development from different types of land. Promoting the development of rural tourism industry with rural land will also be a key area for future research.”**
